# RNA-seq Analysis of Salt-Stressed *Versus* Non Salt-Stressed Transcriptomes of *Chenopodium quinoa* Landrace R49

**DOI:** 10.3390/genes10121042

**Published:** 2019-12-16

**Authors:** Karina B. Ruiz, Jonathan Maldonado, Stefania Biondi, Herman Silva

**Affiliations:** 1 Departamento de Agricultura del Desierto, Facultad de Recursos Naturales, Universidad Arturo Prat, Campus Huayquique, Av. Arturo Prat S/N, Iquique 1100000, Chile; 2 Departamento de Producción Agrícola, Laboratorio de Genómica Funcional y Bioinformática, Universidad de Chile, Av. Santa Rosa 11315, Santiago 8820808, Chile; jomaldon@gmail.com; 3 Dipartimento di Scienze Biologiche, Geologiche e Ambientali, Università di Bologna, Via Irnerio 42, 40126 Bologna, Italy; stefania.biondi@unibo.it

**Keywords:** ethylene-related genes, gene expression, halotolerant crop, salinity, transcriptome

## Abstract

Quinoa (*Chenopodium quinoa* Willd.), a model halophytic crop species, was used to shed light on salt tolerance mechanisms at the transcriptomic level. An RNA-sequencing analysis of genotype R49 at an early vegetative stage was performed by Illumina paired-ends method comparing high salinity and control conditions in a time-course pot experiment. Genome-wide transcriptional salt-induced changes and expression profiling of relevant salt-responsive genes in plants treated or not with 300 mM NaCl were analyzed after 1 h and 5 days. We obtained up to 49 million pairs of short reads with an average length of 101 bp, identifying a total of 2416 differentially expressed genes (DEGs) based on the treatment and time of sampling. In salt-treated vs. control plants, the total number of up-regulated and down-regulated genes was 945 and 1471, respectively. The number of DEGs was higher at 5 days than at 1 h after salt treatment, as reflected in the number of transcription factors, which increased with time. We report a strong transcriptional reprogramming of genes involved in biological processes like oxidation-reduction, response to stress and response to abscisic acid (ABA), and cell wall organization. Transcript analyses by real-time RT- qPCR supported the RNA-seq results and shed light on the contribution of roots and shoots to the overall transcriptional response. In addition, it revealed a time-dependent response in the expression of the analyzed DEGs, including a quick (within 1 h) response for some genes, suggesting a “stress-anticipatory preparedness” in this highly salt-tolerant genotype.

## 1. Introduction

Quinoa (*Chenopodium quinoa Willd*.) is an ancient crop native to South America (around Lake Titicaca in Bolivia and Peru) that is receiving attention worldwide due to its high nutritional value [1] and content in health-promoting compounds [2]. In addition, it is highly resistant to environmental stresses [3,4]. In 2013, the United Nations Food and Agriculture Organization (FAO) recognized quinoa as a key crop for food security and sustainability under a scenario of global change [5,6]. Thus, interest in the crop is still growing, mainly due to increasing water scarcity and soil salinization on a global scale. The production of quinoa in Chile is low as compared to top quinoa-producing countries, namely Peru and Bolivia. This is in part due to less competitiveness in crop management and market access, but also to the limited knowledge of growers and consumers about Chilean quinoa grain and its particular nutritional characteristics [7]. 

Despite a wealth of information regarding salinity responses and possible tolerance mechanisms in quinoa [8,9], studies at the transcriptional level are still relatively scarce. Changes in transcript abundance of the ion transporter *CqSOS1* under NaCl treatment were reported by Maughan et al. [10]; subsequently, the same gene and another gene (*CqNHX*) involved in ion homeostasis were comparatively analyzed in several Chilean landraces originating from contrasting habitats [11]. More recently, the expression of 24 different genes belonging to a number of categories [growth-related, ion homeostasis, transcription factors, abscisic acid (ABA) perception, and biosynthesis, and polyamine biosynthesis], and other salt-responsive genes were analyzed under salt stress (300 mM NaCl) in two Chilean landraces belonging to different ecotypes, i.e., the “*salares*” and the “coastal-lowlands” [8]. Today, the availability of the quinoa genome and high-throughput genomic methods have provided new tools for addressing the complexity associated with stress tolerance in this species [12,13]. To identify candidate salt tolerance genes, Schmöckel et al. [9] focused their study on transmembrane proteins, using a multifaceted approach integrating RNA-seq with single nucleotide polymorphism (SNP) analyses. They identified 219 candidate genes having more than one predicted transmembrane domain that responded to salinity and was specific to or over-represented in quinoa as compared to other Amaranthaceae species. 

We have chosen the drought- and salt-tolerant Chilean landrace R49 for better understanding its salt tolerance mechanisms through exploring the plant’s transcriptome under saline and non-saline conditions. This landrace belongs to the “*salares*” ecotype [14] and originates from the arid northern part of the country (Chilean *altiplano*). Recently, a genome-wide transcriptomic study by RNA-seq was carried out in R49 under drought stress [15]. In order to complement current knowledge on stress-induced transcriptional responses in this quinoa genotype, the present RNA-seq analysis was conducted after exposing R49 plants to high salinity. The novelty of the present work consists in the fact that this is the first time that a full transcriptomic analysis is conducted in a quinoa genotype belonging to the “*salares*” ecotype under saline conditions. The transcriptomic analysis conducted by Schmöckel et al. [9] was aimed at identifying transmembrane proteins associated with salinity tolerance in the “coastal” ecotype reference genome P614886 [13]. Moreover, as opposed to similar studies on quinoa conducted previously [9,15,16], we analyzed the transcriptome at two time-points (1 h and 120 h); in particular, the short-term salinity-induced transcriptional response after 1 h of exposure to 300 mM NaCl has not been previously considered. In spite of its high salt tolerance, early vegetative growth stages in quinoa are quite sensitive to stress conditions [8,17], thus the study was performed on seedlings.

Based on our RNA-seq results and on previous studies regarding genes that are known to be induced by salinity in glycophytic and halophytic model species [8,18,19,20], 10 genes were selected for expression profiling by real-time RT- qPCR analysis. The analyzed genes fall in three main categories: (i) stress-related genes that have been previously indicated as markers in salt-tolerant species, i.e., *carotenoid cleavage dioxygenase 4* (*CarD4*), *lipoxygenase* (*LOX*), *leucoanthocyanidin dioxygenase* (*LDOX*), *rd22-like protein* (*RD22*), and *δ-1-pyrroline 5-carboxylase synthetase* (*P5CS*); (ii) ethylene (ET) perception-related (*Ethylene Receptor1*, *ETR1*) and biosynthetic (*1-aminocyclopropane-1-carboxylic acid synthase*, *ACS; 1-aminocyclopropane-1-carboxylic acid synthase 2*, *ACS2; 1-aminocyclopropane-1-carboxylic acid oxidase-like protein*, *ACO1*) genes, and (iii) a growth-related gene, *β-expansin* (*β-EXP*). Results shed new light on salt tolerance mechanisms at the molecular level in a highly halotolerant crop species.

## 2. Materials and Methods 

### 2.1. Plant Material 

Seeds of *Chenopodium quinoa* landrace R49 were provided by the seed bank of INIA-Vicuña, Chile. Approximately 200 seeds were pre-sterilized in ethanol/water 70% (v/v) for 5 min, followed by 10% hypochlorite commercial solution for 5 min and then subjected to several rinses in autoclaved water. The seeds were vernalized for 12 h at 4 °C and then directly sown in plastic pots containing perlite. They were periodically irrigated with water supplemented with fertilizer (N:P:K 10:10:27; 0.4 g L^−1^; Phostrogen Bayer Garden, Cambridge, UK) and grown under growth chamber conditions (photoperiod 16 h light/8 h dark at a temperature of 21 °C). 

**Figure 1:**
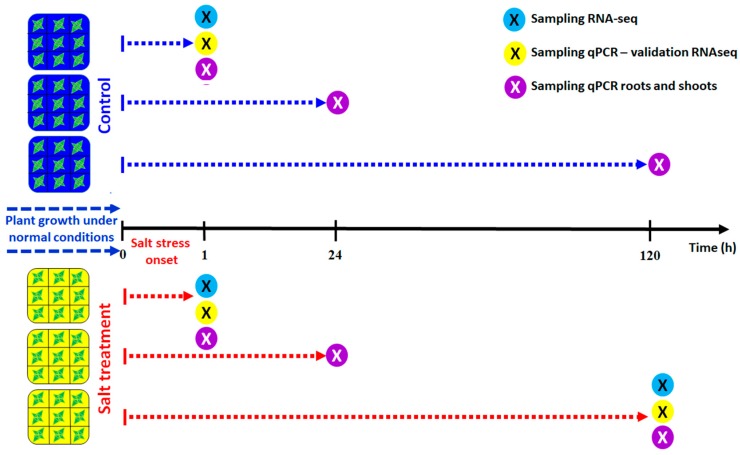
Experimental design and sampling times from the start of salt treatment for RNA-seq and real-time RT-qPCR analyses.

When seedlings had 6 fully-expanded leaves (*ca*. 40-days old), 108 individuals were selected and separated into two independent experiments with 54 individuals each. Salt stress was imposed by irrigating with Phostrogen solution added with 300 mM NaCl (treated plants) or not (control plants; 0 mM NaCl). Control and NaCl-treated plants (27 individuals each) were collected at 3 different times: 1, 24, and 120 h after treatment (AT). At each time point, a total of 9 individuals were subdivided into 3 biological replicates (i.e., each biological replicate was composed of 3 plants that were pooled together), from which roots and shoots were collected separately, weighed, and frozen in liquid nitrogen (Figure 1). Dry biomass of roots and shoots was measured, indicating that the 2 independent experiments gave similar results. Therefore, for the transcriptomic analysis, we worked with material from a single experiment. Dry weight (DW) was determined in freeze-dried samples.

### 2.2. RNA Extraction

Total RNA was extracted according to Chang et al. [21]. RNA yield and purity were checked by means of UV absorption spectra, whereas RNA integrity was determined by electrophoresis on an agarose gel. The concentration of the RNA extracted was evaluated using the NanoDrop spectrophotometer (Thermo Scientific-1000, Thermo Fisher Scientific, Wilmington, DE, USA). DNA was removed using the TURBO DNA-free™ kit (Life Technologies, Carlsbad, CA, USA) from 10 μg aliquots of total RNA, allowing us to proceed with a high-quality RNA.

**Figure 2:**
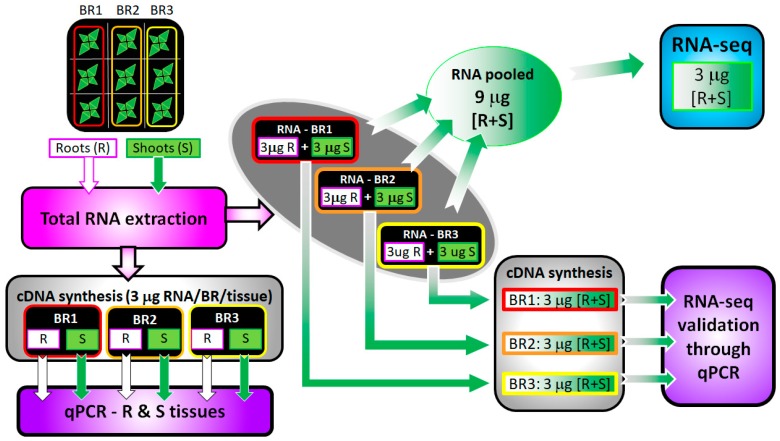
Scheme of RNA extraction from the roots (white square) and shoots (green square) and their utilization, either pooled or not, for RNA-seq and real-time RT-qPCR studies. BR: Biological replicate; R: Roots; S: Shoots. See Material and Methods, Section 2.1 and Section 2.2.

RNA was extracted separately from roots and shoots (using 3–5 samples of 0.1 g fresh weight each). For each biological replicate (i.e., group of 3 plants per treatment and per time point), 3.0 µg of RNA extracted from roots and 3.0 µg from shoots, were pooled to obtain 6 µg of total RNA (roots + shoots; R + S). From this 6.0 µg, 3.0 µg was taken from each biological replicate and combined (giving a total of 9.0 µg) for the RNA-seq analysis; thus, the RNA represented the average of 9 individuals. For the validation of some DEGs by real-time RT-qPCR analysis, 3.0 µg from each biological replicate (roots + shoots; R + S) were used for cDNA synthesis (*n* = 3). The specific contribution of each organ was also studied by real-time RT-qPCR analysis. In this case, the cDNA was synthesized from 3.0 µg total RNA extracted from roots and shoots separately and for each of the 3 biological replicates (*n* = 3) (Figure 2).

### 2.3. Library Preparation and RNA Sequencing 

The following 3 samples were prepared for the transcriptomic analysis: (i) roots and shoots under control conditions, collected 1 h after the salt treatment was initiated in salt-treated samples; (ii) roots and shoots from plants irrigated with 300 mM NaCl and collected 1 h AT; (iii) roots and shoots from plants irrigated with 300 mM NaCl and collected 120 h AT. For RNA sequencing, three samples of 3.0 µg each of RNA purified from roots and shoots of the above-described samples were mixed together. RNA samples with integrity values ≥ 7.0 were used for library preparation. The RNA library was prepared using the Illumina TruSeq protocol (Macrogen, Seoul, South Korea) after constructing a TruSeq mRNA library for paired-end application with an insertion length of 550 bp. The transcriptome sequencing for each sample was carried out with the Illumina HiSeq2000 platform (Illumina, San Diego, CA, USA). 

### 2.4. RNA Sequence Analysis

Trimming and mapping of the sequence reads were performed using the CLC Genome Workbench version 10.1.1 (CLC Bio: CLC genomics workbench [http://www.clcbio.com]). Trimming was based on the following parameters: Q ≥ 20; no more than two ambiguities; final read length ≥ 50 bp. Then, reads of each sequenced library were mapped against the predicted coding sequences of the *C. quinoa* genome (44,776 predicted genes, [13]) using the following parameters: Similarity = 0.9; length fraction = 0.6; insertion/deletion cost = 3; mismatch cost = 3, and unspecific match limit = 10. Relative transcript abundance was obtained as the unique number of reads mapped to each gene. The transcript abundance in these datasets was compared using a *Z*-Test [22]. This test compares counts by considering the proportions that make up the total sum of counts in each sample, correcting the data for sample size. For visual inspection, original over/under-represented values were transformed by the Log10 method and then normalized using the quantile method [23,24].

### 2.5. Functional Annotation of Reference Transcriptome and Gene Ontology Over-Representation Analysis

Functional annotation of *C. quinoa* reference transcripts [13,15] was performed by BLAST2GO software [25] as a plugin of the CLC Genomics Workbench software version 10.1.1 (plugin version 1.1.0). First, a BLASTx search was performed against the NCBI NR database [26] with an e-value cut-off of 1e-6 and HSP length cut-off of 33. Then, an INTERPROSCAN analysis [27] was performed with BLAST2GO default parameters. Finally, the data from BLAST searches, INTERPROSCAN terms, enzyme classification codes (EC), and metabolic pathways (KEGG, Kyoto Encyclopedia of Genes and Genomes) were merged in gene ontology (GO) terms for a wide functional range cover in annotation [26]. The BLAST2GO program defaults were used in all mapping and annotation steps, and the false discovery rate (FDR) cut-off was set to 0.05% probability level. GO over-representation analysis was performed with BLAST2GO tools with a false discovery rate (FDR) cut-off of 0.05% probability.

### 2.6. Real-time Quantitative Reverse Transcription-Polymerase Chain Reaction (RT-qPCR)

In order to validate the RNA-seq data and examine the relative contribution of roots and shoots to the transcriptional response, 10 differentially expressed genes (DEGs) with a two-fold change and an FDR *p*-value < 0.05 were selected for real-time RT-qPCR analysis. Total RNA from roots and shoots was extracted from control and salt-treated samples collected at 1, 24, and 120 h AT (Figure 1) and used to quantify gene expression in each organ (Figure 2). Elongation Factor1a (*CqElF1α*) was used as the reference gene to normalize and estimate up- or down-regulation of the target genes. Transcript levels are shown as mean normalized expression (MNE). All primers are listed in the supplementary material (Table S1). The expression analysis was performed by Q-gene software, as previously described by Ruiz et al. [8]. Data are means (± standard deviation) of 3 biological replicates.

### 2.7. Statistical Analysis

Two independent experiments were conducted; each experiment comprised 54 individuals, divided into 2 treatments (0 and 300 mM NaCl) and 3 collection times (1, 24, and 120 h) resulting in 9 control and 9 salt-treated plants per time point. These were collected (roots and shoots separately), pooled in 3 groups of 3 individuals (i.e., 3 biological replicates) and processed for RNA extraction. A two-way analysis of variance (ANOVA), followed by a Tukey’s post-test, was performed to statistically evaluate significant differences (*p* ≤ 0.05) in root and shoot DW determinations and in the real-time RT-qPCR dataset (InfoStat software [28]). For RNA-seq statistical purposes, we discarded the use of linear models due to the lack of replicates, a crucial factor for dispersion estimation on these methods. Although it is possible to use those methods without replicates, it is not recommended, therefore, we decided to use the Z-test approximation that is designed for such cases.

## 3. Results

### 3.1. Phenotype Response and Transcriptome Sequencing

**Figure 3:**
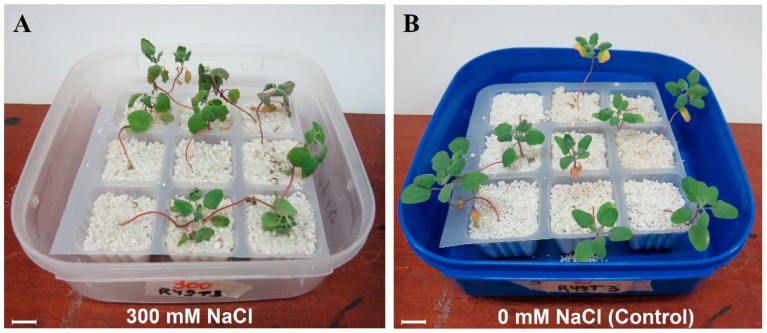
Forty-five-day-old quinoa plants grown in perlite used for RNA-seq and real-time RT-qPCR analyses (**A**) plants treated with 300 mM NaCl and (**B**) controls (0 mM NaCl). Scale bar = 1 cm.

Although salt-treated individuals exhibited no differences in biomass or plant height compared with controls, leaves of salt-treated plants at 120 h AT appeared wilted (Figure 3).

The three RNA-seq samples generated approximately 45 to 49 million pairs of short reads, with an average length of 101 bp, and a similar quantity of mapped reads that was ca. 72% of the total read count (Table 1).

**Figure 4:**
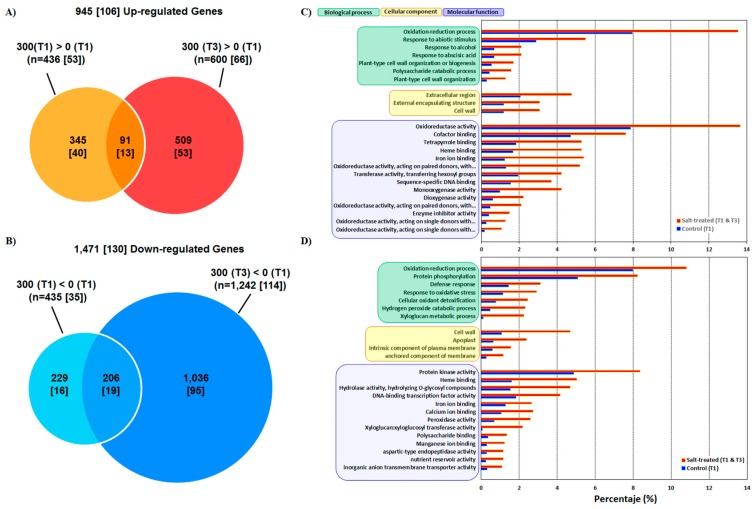
Venn diagrams showing the number of differentially expressed genes (DEGs) (*n* = 2416) in response to 300 mM NaCl at 1 h (T1) and 120 h (T3) after treatment and their overlaps. The number in square brackets indicates the number of genes encoding for transcription factors. (**A**) Genes induced by salt at 1 h and 120 h AT (after treatment) or both. (**B**) Genes down-regulated by salt at 1 h and 120 h AT or both. The size of each group is proportional to the total number of DEGs. (**C**,**D**) Functional categories and biological processes of genes that were induced (**C**) or repressed (**D**) by 300 mM NaCl at T1 and/or T3. Red bars: Salt-treated; blue bars: Controls.

Implementing a model based on read counts and using the cut-off value of two-fold change and an FDR *p*-value <0.05, identified a total of 2416 DEGs based on the salt-stress treatment and time of sampling (Figure 4). The total number of up-regulated genes in salt-treated vs. control plants at both sampling times was 945, of which 91 were shared, including 13 transcription factors (TFs; Figure 4A, Table 2). The number of up-regulated genes was higher in 300 mM NaCl-treated plants at 120 h AT than at 1 h AT (600 and 436 contigs, respectively), and this was also reflected in the number of TFs up-regulated in both groups, which increased slightly (Table 2).

In a total population of 1471 down-regulated genes (Figure 4B), 206 were shared between the two sampling times, including 19 TFs, several of which are linked to the ET response pathway (Table 2). The number of down-regulated genes was about three-fold higher in 300 mM NaCl-treated plants at 120 h AT than at 1 h AT (1036 and 229 contigs, respectively). This was also reflected in the number of down-regulated TFs that registered a six-fold increase (Table 2). 

A subset of 36 DEGs, listed in Table 2, are relevant to several processes linked to regulation of transcription, defense and stress responses, cell wall organization, and ABA metabolism/response. In regards to ET signaling, down-regulation of several *Ethylene-Responsive Factor* (*ERF*) genes was detected in response to salt treatment. Up-regulated genes in the “transcriptional regulation” category were those coding for a TF with high similarity to the *Arabidopsis* NAC domain-containing protein 72, two homeobox-leucine zipper (HAT 5 and ATHB-12-like) proteins, and a heat stress TF (C-1). Moreover, in the “cell wall organization or biogenesis” category, mRNA levels of a polygalacturonase and an expansin (A10) were substantially increased, while several xyloglucan endotransglucosylase genes and an expansin A7-like gene were decreased (Table 2). Salinity also affected the transcription of some stress-related genes; up-regulation of a putative 4-hydroxy-4 methyl-2 oxoglutarate aldolase 3, a universal stress protein A-like protein, MLP-like protein 43, and an 18-kDa seed maturation protein-like, encoding a chaperonin, was observed in salt-treated plants. The expression of genes coding for nodulin-related protein 1-like, a protein phosphatase 2C 37-like, and the ABA receptor PYL9 was stimulated as well. Another evident effect was the transcriptional repression of acidic chitinase and the LRR receptor-like serine/threonine protein kinase FLS2, both of which are involved in defense responses.

### 3.2. Functional Annotation and GO

Results of the functional annotation are shown in Figure 4C,D and File S1. The GO grouped up-regulated genes in three main categories: (1) biological processes, (2) molecular function, and (3) cellular components. A large number of genes that were over-represented under salinity were identified in the biological processes “oxidation-reduction process” and “response to stress” (14% and 5%, respectively; Figure 4C and File S2). Other highly represented biological processes were “response to ABA” (2.5%) and cell wall organization (“plant-type cell wall organization or biogenesis”, “polysaccharide catabolic process”, and “plant-type cell wall organization” for a total of ~6%). Amongst the down-regulated genes (Figure 4D and File S3), the most enriched subcategory related to biological processes was “oxidation-reduction” (~11%) followed by “protein phosphorylation” (8%), “defense response” (3%), and “response to oxidative stress” (3%). 

In the molecular function category, it was found that ca. 14% of up-regulated genes were associated with oxidoreductase activity, followed by genes with binding functions to cofactors (7%), heme groups (~5%), and iron (5%). Analysis of molecular functions that were affected by salinity also showed a large number of genes related to “protein kinase activity” (~8% of the down-regulated gene population), while about 18% of genes were represented in the more general subcategory of “protein binding” that groups heme-, DNA-, TF-, and several types of ion-binding proteins. 

Regarding the cellular components category, “cell wall” (4%), together with “apoplast “(3%) were over-represented under salt treatment. Interestingly, “cell wall”, “extracellular region”, and “external encapsulating structure” were the most represented subcategories in the cellular components’ category (~13%).

### 3.3. RNA-seq Validation by Real-Time RT-qPCR

To confirm the data obtained by RNA-seq, its assembly, and the analysis made to identify up- and down-regulated genes, the expression of selected DEGs from three different functional categories was assessed by real-time RT-qPCR (information regarding target genes is shown in Table S1). To allow comparisons between RNA-seq and real-time RT-qPCR results, the expression value of a given gene, in both cases, was normalized as the ratio against the expression value of the housekeeping gene *ElF1*α (AUR62027947-RA; File S4).

**Figure 5:**
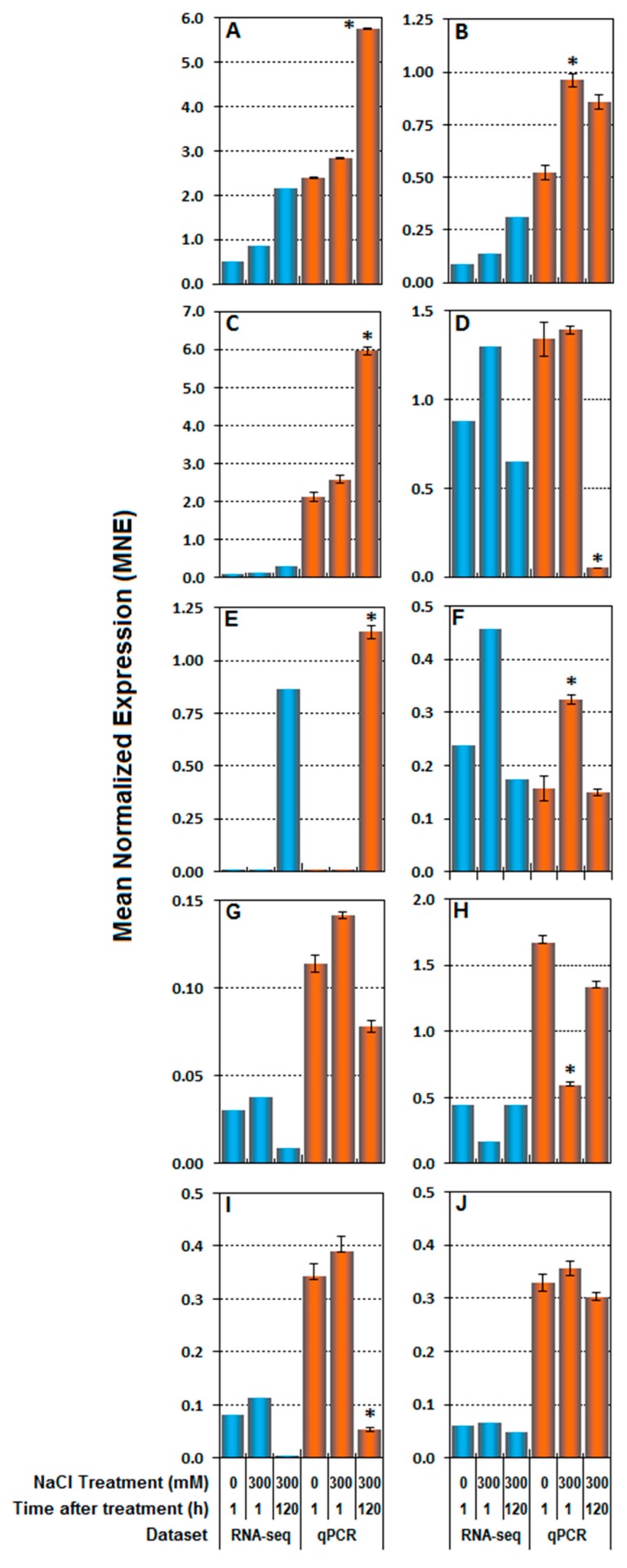
Validation of RNA-seq results by real-time RT-qPCR. The fragments per kilobase per million mapped reads (FPKM) values were calculated from globally normalized RNA-seq data (blue bars). Mean normalized expression (MNE) was obtained by RT-qPCR analysis (orange bars). Both transcript analyses consider 10 genes selected from the RNA-seq dataset to represent different functional categories. Stress-responsive genes: *CarD4* (**A**), *RD22* (**B**), *P5CS* (**C**), *LOX* (**D**), *L-DOX* (**E**); ethylene-related genes: *ETR1* (**F**), *ACS1* (**G**), *ACS2* (**H**), *ACO1* (**I**); growth-related gene: *β-EXP* (**J**). The expression data were normalized to *Elongation Factor1α* (*CqElF1α*) and correspond to means of three biological replicates ± SE. Asterisks indicate significant differences (*p* < 0.05) relative to the control for each set of data.

The expression levels of *CarD4* in salt-treated samples at 120 h AT was two-fold higher than at 1 h AT and in controls. In this case, both RNA-seq and real-time RT-qPCR results showed the same expression profile (Figure 5A). *RD22* transcript amounts increased, but not significantly in salt-treated samples compared to corresponding controls, while the RNA-seq dataset indicated an increase at 120 h AT (Figure 5B). In both RNA-seq and real-time RT-qPCR datasets, *P5CS* transcripts showed a similar profile in the three samples, with a significant three-fold increase at 120 h AT (Figure 5C). *LOX* also showed a similar expression profile in RNA-seq and real-time RT-qPCR datasets with a significant decrease in salt-treated plants as compared with controls at 120 h AT (Figure 5D), while *LDOX* transcript abundance was significantly higher in salt-treated plants collected at 120 h AT in both datasets (Figure 5E). 

We also investigated the ET pathway, which is associated with plant growth and development as well as stress responses. The core of this pathway consisted of two main catalytic enzymes, *ACS*1 and 2, and *ACO*, together with one gene involved in ET perception (*ETR1*). *ETR1* transcript amount showed similar expression profiles in both datasets, with a significant increased 1 h AT in the salt-treated samples (Figure 5F). Both *ACS1* and *ACS2* showed a similar transcript profile in the two datasets; *ACS1* mRNA content decreased at 120 h AT (Figure 5G), whereas *ACS2* transcript amount decreased sharply 1 h AT relative to controls (Figure 5H). *ACO* expression was also significantly down-regulated at 120 h AT compared to controls in both datasets (Figure 5I).

Finally, transcript content of *β-EXP* showed the same trend in both RNA-seq and real-time RT-qPCR analyses, with salinity exerting no significant effects (Figure 5J). 

### 3.4. Expression Levels in Roots vs. Shoots

**Figure 6:**
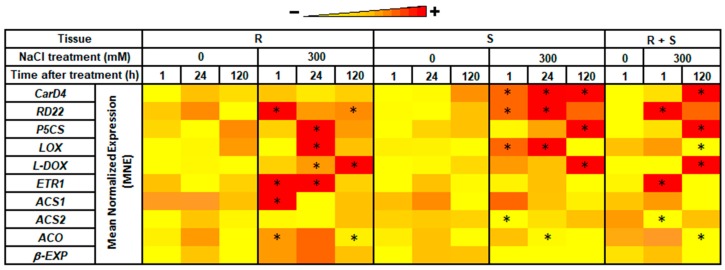
Heatmap representation of the expression profiles (mean normalized expression, MNE) of 10 selected genes after different times (1, 24, and 120 h) of exposure to 0 or 300 mM NaCl. RNA was extracted from roots (R) and shoots (S) separately or pooled prior to cDNA synthesis (R + S). Darker and lighter color shadings represent relatively higher and lower expression levels, respectively. Asterisks indicate significant differences (*p* < 0.05) relative to the control at 1 h.

In order to verify the contribution of the single organ to changes in gene expression, real-time RT-qPCR analyses were also performed in roots and shoots separately. The same 10 genes used for the validation of the RNAseq whose transcript levels showed a different expression profile in the RNA-seq analysis, were selected to draw up a heatmap of their time-course changes in MNE under salinity (Figure 6). (1) Stress-related genes: *CarD4* was highly expressed in shoots under salinity, with maximum expression occurring late (at 24 and 120 h AT), while in roots, *CarD4* transcript content was low and stable over time. The expression profile of *RD22* exhibited the highest expression very early after transfer to saline medium, i.e., at 1 and 24 h AT in roots and shoots, respectively. *P5CS* was a highly expressed gene, especially at 120 h in shoots, while in roots, its expression peaked at 24 h AT. In both tissues, *LOX* showed a significant down-regulation at 1 h and 120 h AT, while a peak of expression was observed at 24 h AT. *L-DOX* showed a maximum expression at 120 h AT in both organs. (2) ET-related genes: in roots, *ETR1* transcript levels showed a significant early increase (1 and 24 h AT), while at 120 h AT, it returned to control values; in shoots, *ETR1* was generally down-regulated. *ACS1* transcript content was up-regulated very early (1 h AT) in roots, while *ACS2* expression was more constant over time in both organs. *ACO1* expression increased early (1 h and 24 h) in roots after transfer to saline medium; it was significantly down-regulated at 120 h AT in both tissues. (3) Growth-related gene: *β-EXP* expression was relatively low as compared with other genes and higher in roots than in shoots. The expression of *β-EXP* in salt-treated roots was significantly up-regulated only at 1 h AT; in shoots, its expression was always below or similar to control values at all sampling times.

## 4. Discussion

### 4.1. Salinity Induces Transcriptome-Wide Changes in Quinoa

The Chilean landrace selected for this study, R49, belongs to the “*salares*” ecotype. It grows in salt flat regions of the Andean *altiplano*, at altitudes of 3500–3900 m a.s.l. R49 has a long vegetative phase (ca. 180 days), and it is locally used by quinoa growers due to its high tolerance to frost [29]. Seedling establishment and the early vegetative stage are described as the most sensitive growth phases due to the need for suitable environmental conditions, such as water availability (i.e., seasonal rains), that favor root growth, thus enabling the plant to avoid the high salinity present in the upper soil layers [30,31].

Previously, we showed that, under a prolonged exposure time (45 days), 300 mM NaCl inhibited root and shoot growth in R49 [17]. In the present work, plants treated with the same concentration of salt did not display significant growth inhibition after 1 week of treatment, even though some visual symptoms of toxicity (wilting) were observed on the leaves. A similar lack of severe growth inhibition was reported in several other quinoa genotypes having a similar origin as R49, i.e., *Real* and *Ollague* [9]. In the case of this particular ecotype, its extremely slow growth rate could contribute to better tolerating stress as described by Raney et al. [16] for *Ollague* exposed to drought conditions. This “trade-off” between growth and resistance was previously observed in a comparison between the “*salares*” genotype *Utusaya* and the Danish-bred cv. *Titicaca* [32]. 

Sequencing of the quinoa genome [13] combined with three RNA-seq analyses [9,15,16] have increased the availability of genetic information for this halophytic seed-producing crop. The two former reports are on quinoa subjected to drought stress, while the latter considered the reference genome PI614886 [13], belonging to the “coastal” ecotype and originating from Maule (central Chile), under salt stress. Raney et al. [16] identified 462 DEGs in root tissue of *Ollague*, a Chilean genotype belonging, like R49, to the “*salares*” ecotype, while Morales et al. [15] found 2456 DEGs after drought stress treatment in combined roots and shoots of R49. In PI614886 subjected to 300 mM NaCl, Schmöckel et al. [9] found a total of 5811 DEGs in roots and shoots (analyzed separately), compared with 2416 DEGs reported in the present study that considers roots and shoots pooled together. Conversely, the number of salt-induced DEGs presently observed is similar to that found by Morales et al. [15] for the same landrace under drought stress. 

In several studies, the DEGs reported under salt stress in glycophytes were more abundant than in halophytes [18,33,34,35]. The lower number of DEGs found by us for R49 (“*salares*” ecotype) as compared with the less salt-tolerant PI614886 (“coastal” ecotype) suggests that the number of DEGs under salt stress may be inversely proportional to tolerance.

The above-mentioned RNA-seq analyses in quinoa were done only at one time-point (4 days, 7 days, or 4 weeks), while in the present work, we also considered the time factor and, for the first time, investigated the very short-term response (1 h AT). We show here that DEGs increased with exposure time (from 1 h to 120 h) and that down-regulated contigs increased more than the up-regulated ones. Thus, at 120 h AT, the ratio between up- and down-regulated genes (509 vs. 1036 contigs, respectively) declined by about twice. A similar increase with time in the number of down-regulated genes was observed in *Arabidopsis* (at 2 vs. 10 h) by Matsui et al. [33], by Gong et al. [19] in *Arabidopsis* and *Thelungiella*, in chickpea by Mantri et al. [36], in rice by Wang et al. [37] and even in a xerohalophyte, *Zygophyllum xanthoxylum* [38]. Moreover, several studies reported that salt-tolerant genotypes down-regulate fewer genes than salt-sensitive ones [19,34,38,39]. In quinoa subjected to salt stress for 7 days, Schmöckel et al. [9] reported 622 DEGs in both root and shoot tissues. In this population of shared genes, the down-regulated transcripts were more abundant than up-regulated ones. An opposite pattern was reported in quinoa plants subjected to drought stress [15]. The reason for the increased number of down-regulated genes with increasing exposure time, as presently observed in quinoa under high salinity, could be due to a tight control and efficiency in shutting down the transcriptional process, mainly for those genes associated to oxidative activities and to the cell wall compartment, that could be constitutively turned on. In a comparative study between salt-sensitive *Arabidopsis* and its salt-tolerant relative *Thelungiella*, Gong et al. [19] showed that some gene clusters were differently regulated under salinity stress. Those that were up-regulated in *Arabidopsis* but down-regulated in *Thellungiella* were mainly general defense-related genes and transcription factors, together with genes with protein translation–initiation functions, suggesting that *Arabidopsis* initiates a global defense response that requires the presence of newly synthesized proteins. They proposed that *Thellungiella* attempts to conserve resources and energy under stress conditions. Again, this form of “trade-off” may be part of the “halophyte’s way” to deal with high salinity.

### 4.2. Comprehensive Analysis of DEGs induced by Salt Stress and Biological Insight

In the present study, a total of 2416 salinity responsive DEGs were identified. These fell into three main categories involving a series of biological processes, mostly linked to oxidation-reduction processes, responses to abiotic stimulus, and protein phosphorylation followed by molecular functions (mainly represented by oxidoreductase activity, cofactor-binding, and protein kinase activity). The cellular components category was mainly enriched in genes related to the extracellular region and cell wall components. 

In the same genotype, R49, subjected to drought stress, Morales et al. [15] found that the most represented categories were those related to biological processes, namely primary and organic substance metabolic processes, followed by the category associated to molecular functions, represented by nucleus and plastids. In the cellular component’s category, the most enriched subcategories were those related to “organic cyclic and heterocyclic compound binding”. In the RNA-seq analysis of *Ollague*, Raney et al. [16] found that under drought treatment, transferase activity, metal ion binding, cation binding, protein binding, and oxidoreductase activity were the major activities indicated in the molecular function category, while cellular metabolic process, primary metabolic process, response to stress, and response to abiotic stimulus were the major biological processes. In the RNA-seq study with the reference genome PI614886 subjected to salinity stress, Schmöckel et al. [9] found an enrichment of genes involved in catalytic activity, suggesting that the expression of numerous enzymes increases in response to salt. In the present work, a similar pattern of enriched categories was found in R49 under high salinity. Our transcriptomic analysis of R49 also revealed that a large number of DEGs belonged to the biological processes subcategory “oxidation-reduction process”. This may confirm previous studies reporting a constitutive high antioxidant level (tocopherols, phenolic compounds, and other compounds with antioxidant activity) in quinoa seeds [4,40,41]. 

Interestingly, cell wall organization seems to play an important role in R49 salinity responses, as described for garlic [35] and also for the xerohalophyte *Zygophyllum xanthoxylum* [38]; in response to salt stress, the most enriched subcategories were related to peroxidase activity and lignin formation. In fact, in the categories of biological processes and cellular components, highly represented DEGs were those related to cell wall organization or biogenesis, polysaccharide catabolic process, and cell wall organization together with the extracellular region, apoplast, plasma membrane components, and cell wall-related structures. Indeed, a total of 65 cell wall-related genes were found that were differentially expressed during salt stress, supporting the idea that these may play a crucial role in adaptation to salinity. The cell wall is highly susceptible to salt stress and other biotic and abiotic stresses [42,43]. Salinity usually results in cell wall structure and extensibility alterations as a consequence of changes in levels of lignins, pectins, celluloses, and hemicelluloses [35]. Cell wall extensibility is a vital factor for maintaining normal turgor pressure under salt stress in both salt-sensitive [44,45] and salt-tolerant species [37]. The decline in growth at supra-optimal salinity levels may occur as a result of a decline in turgor [46], consequent upon high concentrations of ions in the apoplast [47], or a change in cell wall elasticity [48].

The expression of some expansin isoforms is correlated with growth, and the external application of expansins can stimulate cell expansion in vivo in several plant systems due to wall loosening [49]. The involvement of expansins in abiotic stress responses has also been documented [50]. Buchanan et al. [51] reported a salinity-induced increase in *β-EXP* transcript abundance for *Sorghum bicolor*. Pitann et al. [52] observed that a β-expansin protein decreased under salinity in a salt-sensitive maize cultivar, whereas it was less affected in a salt-tolerant one. Soon after, Geilfus et al. [53] reported that the maintenance of shoot growth in a salt-resistant variety of maize could be related to an unaffected abundance of β-expansin proteins. Thus, the stable expression of the *β-EXP* gene analyzed here, may be associated with the salt-tolerance of R49. It should be pointed out, however, that the *β-EXP* expression pattern observed in the present study was different from that of in vitro-grown seedlings where *β-EXP* transcript abundance was salt-induced [8]. This difference likely depends on the experimental system used (agar plates vs. perlite) as well as plant age (12-day vs. 40-day old), a factor that can influence stress responses in quinoa [54]. 

Several genes associated with biological processes, as identified by the GO analysis, were explored in their expression profile due to their relevance in responses to abiotic stimuli and defense. In particular, five stress-related genes, i.e., *CarD4, RD22, P5CS, LOX,* and *L-DOX*, were selected for real-time RT-qPCR analysis based on changes in the read numbers detected by RNA-seq. 

*CarD4* transcript levels showed the same pattern in the RNA-seq and real-time RT-qPCR analyses. It was highly expressed only 120 h AT (Figure 5A) and significantly up-regulated only in shoots (Figure 6). In higher plants, carotenoid cleavage dioxygenases cleave specific carotenoids, thereby producing a diverse set of apocarotenoids. These apocarotenoids are precursors for the production of some plant hormones); they are also responsible for producing volatile aromatic compounds [55] and compounds involved in color and aroma formation in fruits and flowers [56]. In *Arabidopsis*, 9 CCDs have been classified (CCD1, 4, 7, 8, and NCED2, 3, 5, 6, 9; [57]). CCD7 and 8 were found to be important in the production of strigolactones [58,59], while NCEDs are mainly involved in ABA biosynthesis [60]. The CCD4 family is the largest family of plant CCDs, only present in flowering plants. In saffron, expression of *CsCCD4* was significantly induced by dehydration stress and heat treatment [61]. In a further study, *CsCCD4c* was up-regulated by wounding, heat, and osmotic stress, suggesting the involvement of its apocarotenoid products in the adaptation of saffron to environmental stresses [62]. Its up-regulation in salt-stressed R49 suggests that it also plays a critical role in stress adaptation in quinoa.

The stress-related genes *RD22* and *P5CS* were quite similar in their pattern of expression in the RNA-seq and real-time RT-qPCR analyses (Figure 5B,C). P5CS is the key biosynthetic enzyme in the biosynthesis of proline, a well-known osmoprotectant, while *RD22* encodes for drought-inducible dehydration responsive protein [63,64]. Both transcripts tended to be up-regulated early; however, while *P5CS* maintained the up-regulation until 120 h, *RD22* returned to control levels. A similar expression profile was observed in our earlier study comparing R49 with Villarrica, a landrace belonging to the “coastal-lowlands” ecotype [8], indicating a differential role for *RD22* and *P5CS* in the longer-term adaptive response of the “*salares*” landrace. 

Lipoxygenases (LOXs) are a special class of dioxygenases that catalyze the conversion of polyunsaturated fatty acids and lipids into oxylipins, such as jasmonic acid and methyl jasmonate, having several roles in defense responses to biotic or abiotic stresses [65]. Our results (Figure 5D and Figure 6) show an early induction of *LOX* transcript content that declined at the end of the experiment. A similar pattern for *CaLOX1* expression in response to high salinity was described in *Capsicum annuum* where transcription of the gene was induced at 6 h and gradually declined from 12 to 24 h after NaCl treatment [66,67]. *CaLOX1* was significantly induced in pepper leaves also by bacteria, ethylene, and salicylic acid [66].

The flavonoid biosynthetic pathway is also strongly activated by salinity [68]. Few reports have appeared, however, concerning gene expression and regulation of flavonoid biosynthesis in halophytes and other stress-resistant species. In our transcriptomic study, the gene leucoanthocyanidin dioxygenase (*LDOX*; Figure 5E and Figure 6) was highly expressed, relatively late (120 h AT), in response to salt stress. *LDOX* expression is usually regulated to protect plants against damage from UV irradiation and low temperature [69] and also exhibits significant differences in transcript abundance under salt stress [68]. Zhang et al. [70] reported an increase in *LDOX* transcript levels in *Reaumuria trigyna* under 500 mM NaCl treatment. This enhanced expression under salt stress was attributed to unique physiological characteristics, and molecular regulatory mechanisms evolved in this species in response to the salinized desert environment in which it has been growing for millions of years. 

In many studies on glycophytes, enhanced tolerance to salt stress was shown to be associated with the induction of the ET signaling pathway [71,72,73]. For instance, increased expression of the ERF transcription factor in wheat and tobacco conferred improved tolerance to salinity [74,75], whereas enhanced oxidative stress was detected in the ethylene-insensitive (*ein3-1*) mutant of *Arabidopsis* exposed to salt stress [76]. Yet, the involvement of ET in salinity responses in halophytes has been scarcely characterized [77,78]. As indicated in our real-time RT-qPCR analysis, *ETR1*, a gene coding for one of the ET receptors, was strongly and rapidly induced in response to salt (Figure 5F). By contrast, the ET biosynthetic genes, *ACS1* and *ACO*, were down-regulated, especially in the long term (Figure 5G,I), while *ACS2* was down-regulated only 1 h AT (Figure 5H). Thus, although *ETR1* was up-regulated, the ET biosynthesis pathway was substantially down-regulated. This is coherent with the fact that the ET signaling pathway was activated by salt stress, since ETR1, and other ET receptors, are negative regulators of this pathway, i.e., they are activated when they are not bound to ET. 

As shown in Figure 6, the salt-induced up-regulation of *CarD4* and *RD22* observed when roots and shoots (R + S) were analyzed together was mainly due to enhanced transcript abundance in shoots. The enhanced expression level at 120 h AT of *P5CS* also occurred mainly in shoots, whereas the up-regulation of *L-DOX* occurred both in roots and shoots. By contrast, roots accounted for the up-regulation of *ETR1* registered in salt-treated plants. A transient increase in *ACS1* expression in roots of salt-treated plants was observed, but this was masked in the R + S samples by the lack of effect in shoots. The lower, as compared to controls, transcript abundance of *ACO* in salt-treated plants could be attributed to both organs. Generally speaking, the heat-map shows that stress-related genes were preferentially up-regulated in shoots, while ET-related genes were differentially expressed mainly in the roots and early on after exposure to salt. Most stress-related genes remained switched on up to the end of the experiment. A similar pattern has been described in chickpea treated with salt and drought stresses at different developmental stages [79] and in two cultivars of barley differing in salt stress tolerance [80]. Our time-course analysis of gene expression also revealed a short-term (within 1 h) response to salinity for several genes. In fact, the extremely rapid up-or down-regulation of most genes (at 1 or 24 h) may be indicative of a pre-adaptive response to salinity in halophytes due to a “constitutive over-expression” of stress-related genes even under non-stressful conditions [18,81]. The expression “stress-anticipatory preparedness” was used by Gong and co-workers [19] in their comparative study on stress tolerance mechanisms in *Arabidopsis thaliana* and its halophytic relative *Thellungiella halophila*. More recently, Kazachkova et al. [81] also refer to a “stress ready transcriptome/metabolome” in halophytes. Since quinoa has many ecotypes, varieties, and landraces, further studies are needed to check whether this trait is a generalized phenomenon in this halophytic crop species.

## 5. Conclusions

In this work, we report the first transcriptome analysis of salt-treated quinoa plants of the “*salares*” genotype R49, which is adapted to a particularly extreme habitat, the *altiplano* of northern Chile. The analysis revealed a total of 2416 salt-responsive DEGs when roots and shoots were pooled together. These fell into three main categories, namely biological processes (mostly oxidation-reduction processes, responses to abiotic stimulus, and protein phosphorylation) and molecular functions (e.g., oxidoreductase activity, cofactor-binding, and protein kinase activity), while the cellular components category was mainly enriched in genes related to the cell wall. Indeed, 65 cell wall-related DEGs were identified under high salinity, confirming the notion that expansins and other components affecting cell wall properties are involved in abiotic stress tolerance. Amongst the stress-associated genes analyzed by real-time RT-qPCR, differential expression patterns were observed both in a time- and an organ-dependent manner. Some genes (e.g., *RD22*, *LOX*) were rapidly up-regulated and then returned to control levels, while others (e.g., *P5CS*) remained up-regulated up to 120 h AT, pointing to the importance of time-course experiments. The involvement of the “stress hormone” ET has, up to now, not been investigated in quinoa. We show here, that at least one component of the ET signaling pathway (the ETR1 receptor) was transcriptionally induced by 300 mM NaCl. 

*Future perspectives*: The use of halophytic seed-producing crops to contrast increasing salinization of soil and water sources could contribute to solving the problem of food security in a climate change scenario [82]. Quinoa seems to be a particularly good candidate due to its high adaptability to new agroecosystems, characterized by salty soils and low water and nitrogen availability, and its excellent nutritional properties [5,6,83]. The results of this RNA-seq analysis improves our knowledge of the salt-induced transcriptional changes in a quinoa landrace that grows in a very extreme environment, the salt flats of the Andean *altiplano*. Such knowledge, recorded in the genetic memory of quinoa, will contribute to future breeding efforts, for example, by marker-assisted selection, for quinoa improvement as well as for breeding other crops with increased salt tolerance.

## Figures and Tables

**Table 1:** Summary of *Chenopodium quinoa*, landrace R49 reads mapping against *Chenopodium quinoa* predicted genes.

	(Treatment) Time		
	(0) T1	(300) T1	(300) T3
**Total Bases**	4,971,425,434	4,581,155,576	4,883,948,526
**Read Count**	49,222,034	45,357,976	48,355,926
**GC (%)**	43.34	43.31	43.51
**Average Length (bp)**	101	101	101
**Inputs Reads**	47,543,290	43,853,712	46,700,950
**Mapped Reads**	34,220,702	31,442,862	33,862,731
**Mapped Rate (%)**	71.98	71.7	72.51
**Q20 (%)**	96.59	96.7	96.58
**Q30 (%)**	91.71	91.96	91.68

*Abbreviations*: G, guanine; C, cytosine; Q, quality score, a common measure used to assess the accuracy of a sequencing Illumina HiSeq2000 platform.

**Table 2:** Set of selected genes up- or down-regulated by salt treatments at 1 h and 120 h, T1, and T3, respectively (overlaps in Figure 3).

Gene	Gene Ontology (GO)	Annotation	Kal’s *Z*-test			
			300) T1 > (0) T1		(300) T3 > (0) T1	
			Fold Change	FDR *p*-Value	Fold Change	FDR *p*-Value
AUR62029344-RA	GO:0006355 Regulation of transcription, DNA-templated	NAC domain-containing protein 72	2.68	0.00	2.45	0.00
AUR62026261-RA		homeobox-leucine zipper protein HAT5	2.90	0.00	2.49	0.00
AUR62021507-RA		heat stress transcription factor C-1	4.06	0.00	2.50	0.00
AUR62004541-RA		homeobox-leucine zipper protein ATHB-12-like	4.64	0.00	5.11	0.00
AUR62023642-RA		ethylene-responsive transcription factor ERF014-like	−3.12	0.00	−4.13	0.00
AUR62004723-RA		ethylene-responsive transcription factor ERF014-like	−2.68	0.00	−3.85	0.00
AUR62025525-RA		ethylene-responsive transcription factor ERF071	−2.27	0.00	−4.07	0.00
AUR62032882-RA		ethylene-responsive transcription factor ERF021-like	−6.55	0.01	− ∞	0.00
AUR62032288-RA	GO:0006952 Defense response	acidic chitinase	− ∞	0.00	−103.63	0.00
AUR62027590-RA		LRR receptor-like serine/threonine-protein kinase FLS2	−2.59	0.00	−3.27	0.00
AUR62033289-RA	GO:0006950 Response to stress	putative 4-hydroxy-4-methyl-2-oxoglutarate aldolase 3	2.79	0.00	4.93	0.00
AUR62037323-RA		universal stress protein A-like protein	4.57	0.00	6.98	0.00
AUR62031231-RA		MLP-like protein 43	2.14	0.00	2.37	0.00
AUR62012039-RA		18 kDa seed maturation protein-like	3.82	0.01	14.18	0.00
AUR62021371-RA		putative 4-hydroxy-4-methyl-2-oxoglutarate aldolase 3	4.92	0.00	4.56	0.00
AUR62032222-RA		inhibitor of trypsin and hageman factor-like	−2.81	0.00	−28.13	0.00
AUR62017675-RA		chalcone synthase	−3.16	0.00	−2.70	0.00
AUR62020156-RA		asparagine synthetase [glutamine-hydrolyzing]	−3.70	0.00	−64.32	0.00
AUR62031453-RA		S-type anion channel SLAH2 isoform X3	−2.35	0.00	−3.15	0.00
AUR62014092-RA		peroxidase 5	−2.09	0.03	−6.63	0.00
AUR62009744-RA		peroxidase 57	−2.67	0.00	−10.86	0.00
AUR62030623-RA	GO:0071554 Cell wall organization or biogenesis	expansin A10	4.70	0.00	2.59	0.00
AUR62024921-RA		expansin A10	4.93	0.00	2.90	0.00
AUR62002580-RA		polygalacturonase At1g48100	2.60	0.00	2.37	0.00
AUR62018907-RA		xyloglucan endotransglycosylase/hydrolase	−2.36	0.00	−9.18	0.00
AUR62020893-RA		xyloglucan endotransglucosylase/hydrolase 2-like	−3.08	0.00	−3.77	0.00
AUR62024859-RA		xyloglucan endotransglucosylase/hydrolase protein 22-like	−2.40	0.00	−3.17	0.00
AUR62035069-RA		COBRA-like protein 7	−2.48	0.00	−2.80	0.00
AUR62018602-RA		expansin-A7-like	−3.07	0.05	−3.78	0.01
AUR62018634-RA		Cellulose synthase-like protein D3	−2.45	0.00	−3.84	0.00
AUR62003130-RA	GO:0009688 Abscisic acid biosynthetic process	nodulin-related protein 1-like	2.14	0.00	2.63	0.00
AUR62012136-RA	GO:0009737 Response to abscisic acid	protein phosphatase 2C 37-like	2.10	0.00	2.03	0.00
AUR62023479-RA		abscisic acid receptor PYL9	3.14	0.00	2.10	0.00
AUR62012310-RA		abscisic acid receptor PYL4	−4.31	0.00	−10.00	0.00
AUR62010485-RA		abscisic acid 8’-hydroxylase 1-like	−3.44	0.00	−3.63	0.00
AUR62022950-RA		abscisic acid receptor PYL4	−2.38	0.00	−11.05	0.00

Comparison of DEGs between salt-treated samples up- or down-regulated at a twofold change and false discovery rate (FDR) *p*-value less than 0.05. Kal’s Z-test of proportions was used for the statistical analysis.

## Supplementary Materials

The following are available online at https://www.mdpi.com/2073-4425/10/12/1042/s1, Table S1: List of primers used in this study for real-time RT-qPCR analysis, File S1: Functional annotation of reference transcriptome, File S2: Gene ontology enrichment of functions that belongs to genes over-represented under salinity, File S3: Gene ontology enrichment of functions that belongs to genes down-regulated under salinity, File S4: RNAseq validation by real-time RT-qPCR.
